# Monocyte to Macrophage Differentiation Goes along with Modulation of the Plasmalogen Pattern through Transcriptional Regulation

**DOI:** 10.1371/journal.pone.0094102

**Published:** 2014-04-08

**Authors:** Stefan Wallner, Margot Grandl, Tatiana Konovalova, Alexander Sigrüner, Thomas Kopf, Markus Peer, Evelyn Orsó, Gerhard Liebisch, Gerd Schmitz

**Affiliations:** Institute of Clinical Chemistry and Laboratory Medicine, University Hospital Regensburg, University of Regensburg, Regensburg, Germany; University of Cologne, Germany

## Abstract

**Background:**

Dysregulation of monocyte-macrophage differentiation is a hallmark of vascular and metabolic diseases and associated with persistent low grade inflammation. Plasmalogens represent ether lipids that play a role in diabesity and previous data show diminished plasmalogen levels in obese subjects. We therefore analyzed transcriptomic and lipidomic changes during monocyte-macrophage differentiation *in vitro* using a bioinformatic approach.

**Methods:**

Elutriated monocytes from 13 healthy donors were differentiated *in vitro* to macrophages using rhM-CSF under serum-free conditions. Samples were taken on days 0, 1, 4 and 5 and analyzed for their lipidomic and transcriptomic profiles.

**Results:**

Gene expression analysis showed strong regulation of lipidome-related transcripts. Enzymes involved in fatty acid desaturation and elongation were increasingly expressed, peroxisomal and ER stress related genes were induced. Total plasmalogen levels remained unchanged, while the PE plasmalogen species pattern became more similar to circulating granulocytes, showing decreases in PUFA and increases in MUFA. A partial least squares discriminant analysis (PLS/DA) revealed that PE plasmalogens discriminate the stage of monocyte-derived macrophage differentiation. Partial correlation analysis could predict novel potential key nodes including DOCK1, PDK4, GNPTAB and FAM126A that might be involved in regulating lipid and especially plasmalogen homeostasis during differentiation. An *in silico* transcription analysis of lipid related regulation revealed known motifs such as PPAR-gamma and KLF4 as well as novel candidates such as NFY, RNF96 and Zinc-finger proteins.

**Conclusion:**

Monocyte to macrophage differentiation goes along with profound changes in the lipid-related transcriptome. This leads to an induction of fatty-acid desaturation and elongation. In their PE-plasmalogen profile macrophages become more similar to granulocytes than monocytes, indicating terminal phagocytic differentiation. Therefore PE plasmalogens may represent potential biomarkers for cell activation. For the underlying transcriptional network we were able to predict a range of novel central key nodes and underlying transcription factors using a bioinformatic approach.

## Introduction

Macrophages are key players in innate immunity and play an important role in the development of atherosclerosis and insulin resistance in diabesity [1]. During atherogenesis, modified ApoB containing lipoproteins accumulate in atherosclerotic plaques and lead to chemotaxis and accumulation of monocytes in the subintima [1]. Under the pro-inflammatory influence of the local microenvironment these monocytes terminally differentiate to M1 or M2 macrophages or antigen presenting cells (APC) [2]–[4]. During early lesion growth macrophages develop resistance to apoptosis and oxidative stress, whereas in advanced lesions macrophage death contributes to the formation of a necrotic core [5]. Consequently metabolic syndrome correlates with persistent low grade inflammation as indicated by increased serum levels of IL-6, CRP and fibrinogen [6]. Moreover metabolic overload induces an ER-stress response and leads to the formation of reactive oxygen species (ROS) [7].

Lipids regulate biological processes either locally as membrane components or remotely as signaling molecules. The lipid composition of the plasma membrane determines membrane fluidity but direct lipid-protein interactions also play a role in cellular signaling [8]–[10]. Moreover the release of signaling lipids from intracellular or membrane sources fulfills an important function in inflammatory signaling [11]. In this context especially eicosanoids, sphingosine-1-phosphate and lysophosphatidic acid are worth mentioning. Plasmalogens are a group of lipids that play a role in most of these tasks. In the plasma membrane they regulate membrane fluidity, via their vinyl-ether bond in sn-1 they act as anti-oxidants and in sn-2 position they carry the precursor residues for n-3 and n-6 prostanoid synthesis. Interestingly cleavage of this esther-bound alkyl chain is catalyzed by plasmalogen-selective phospholipase A2 (PLA2) [11]. In circulating monocytes plasmalogens represent around 15% of all cellular lipids [12]. They have also been shown to possess clinically significant correlations to vascular, metabolic and neurodegenerative diseases [13]. For example lower levels of plasmalogens were found in hypertensive patients and during aging in the aorta (even more pronounced in atherosclerotic aortas) [6], [14]. Similarly plasmalogen depletion in red blood cell membranes has been proposed as a marker for oxidative stress and membrane rigidity and was suggested to be predictive for cardiovascular mortality [15]. Under these conditions plasmalogens may exert a scavenger function for reactive oxygen species in membranes, that could play a role during the ER stress response. On the other hand plasmalogen oxidation products such as alpha-hydroxyaldehydes and plasmalogen epoxides were found to accumulate in atherosclerotic lesions [16].

In a previous study we could demonstrate that a SREBP1 dependent induction of monocyte fatty acid synthesis is vital for monocyte-macrophage differentiation [17]. Detailed analysis of the changes in plasmalogen species and the underlying regulation might therefore provide further insight into the role of this specific lipid classe in the differentiation process. Recent advances in mass spectrometry made high-throughput analysis of plasmalogen species feasible for use in research and in the clinical routine laboratory [18]. This technology allows to follow the regulation of individual species and analysis of regulatory patterns that might reflect pathophysiological adaptations in atherosclerotic lesions. The aim of the current study was therefore to analyze in detail the transcriptomic and lipidomic alterations during the course of monocyte-macrophage differentiation and to evaluate the potential of plasmalogens as marker lipids for differentiation of monocytes to inflammatory macrophages. A bioinformatic approach was used to examine the underlying transcriptional regulation and to reverse engineer gene regulatory networks to identify potential novel key nodes.

## Materials and Methods

### Materials

If not otherwise stated all materials including 1α,25-dihydroxyvitamin D_3_ (VitD_3_), all−trans−retinoic acid (ATRA) and ethanol were obtained from Sigma (Germany). Carrier-free macrophage colony-stimulating factor (M-CSF) and granulocyte macrophage colony-stimulating factor (GM-CSF) were both from R&D systems. HPLC grade methanol and chloroform were from Merck (Germany), plasmalogen standards from Avanti Polar Lipids (Alabaster, AL, USA).

### Blood cell isolation and *in vitro* differentiation of human monocytes

Blood samples were obtained from thirteen healthy normolipidemic volunteers recruited from blood donors with apoE3/E3 genotype. Informed consent and approval of the Hospital Ethics Committee were obtained (Universitätsklinikum Regensburg, Ethikkommission der medizinischen Fakultät, proposal 08/119). Donors were fully informed of the possible complications and gave their written consent for the procedure. Blood cells were collected by leukapheresis in a Spectra cell separator (Gambro BCT, CO, USA) followed by subsequent counterflow elutriation as described elsewhere [19]. In brief, cells were elutriated in the following order: platelets, lymphocytes, monocytes and then granulocytes. Aliquots of the different cell fractions were analyzed for cell purity on a BD FACSCanto flow cytometer (Becton Dickinson) using BD FACSDiva Software. Cell numbers were determined on an ADVIA 120 automated cell counter (Siemens Healthcare Diagnostics GmbH, Germany). Elutriated monocytes were then seeded at a concentration of 1×10^6^ cells/ml in serum-free macrophage medium (Invitrogen) that was supplemented with 50 ng/ml of recombinant human macrophage colony-stimulating factor (rhM-CSF, R&D Systems). 1×10^7^ cells per dish were cultured in 10 cm Cell^+^ plastic petri dishes (Sarstedt) for RNA isolation and 2×10^6^ cells per well in Cell^+^ 6-well plates (Sarstedt, USA) for triplicates in mass spectrometry. Incubation was performed at 37°C in a humidified atmosphere containing 5% CO_2_ in an incubator. After washing with PBS, the cell pellets were stored at −80°C. Protein concentrations were measured according to Smith et al. [20] using the BCA Assay from Uptima-Interchim (France) with serial dilutions of bovine serum albumin as standard.

### *In vitro* differentiation of HL-60 cells

HL-60 cells (ATCC; CCL-240) were grown in an incubator at 37°C (95% humidity, 5% CO_2_) in Iscove's Modified Dulbecco's Medium (IMDM) (PAN-Biotech) containing 20% FBS (Biochrom) in polystyrene tissue culture flasks (Sarstedt). Cells were seeded at a density of 0.3×10^6^ cells per ml and 50% of the medium was replaced once per week. Confluent cultures were split 1∶3 to 1∶4 once per week.

Differentiation experiments were performed in Cell^+^ tissue culture flasks (Sarstedt). 1 μM working solutions of Vitamin D_3_ (VitD_3_)and all-trans retinoic acid (ATRA) were prepared in ethanol. Cells were seeded at 0.3×10^6^ cells per ml. Monocytic differentiation was achieved in macrophage serum-free medium (SFM) supplemented with M-CSF (50 ng/ml) and 0.5 μM VitD_3_. Granulocytic differentiation was carried out in macrophage SFM with GM-CSF (10 ng/ml) and 0.5 μM ATRA. Cells grown in the respective medium containing 0,05% ethanol were used as controls. Cells were harvested by scraping with a rubber policeman and centrifugation for 5 min at 500 g at room temperature. They were finally washed once with PBS. Cell number was determined by counting in a Casy automated cell counter (Roche). Differentiation phenotype and viability were monitored by flow cytometry and Pappenheim staining. Experiments were repeated in two biological replicates.

### Gene expression studies

Cells from nine donors were harvested, washed in PBS, resuspended in buffer RLT and RNA was isolated using the RNeasy Mini Kit (Qiagen) according to the manufacturer's instructions. Purity and integrity of the RNA were determined on the Agilent 2100 Bioanalyzer with the RNA 6000 Nano LabChip reagent set (Agilent Technologies). RNA was quantified using a Nanodrop ND-1000 - UV/Vis spectrophotometer (PeqLab).

For gene array analysis we used a modified Agilent 4×44K microarray (014850) containing 205 free positions (Agilent Technologies). To these positions we added 201 probes, corresponding to 119 genes of the lipidome (LipidomicNet). 300 ng of total RNA were labeled with Cy3 using the Agilent Quick-Amp Labeling Kit - 1 color according to the manufacturer's instructions. cRNA was purified with the RNeasy Mini Kit (Qiagen). The amount and labeling efficiency of cRNA was measured with the NanoDrop ND-1000. For hybridization the Agilent Gene Expression Hybridisation Kit was used and arrays were incubated in Agilent SureHyb chambers for 17 hours in a hybridization oven at 65°C while rotating. Washing was performed according to the manufacturer's instructions. Scanning was done with the Agilent G2565CA Microarray Scanner System. The resulting TIFF files were processed with Agilent Feature Extraction software (10.7). Microarray data are available in the ArrayExpress database (www.ebi.ac.uk/arrayexpress) under accession number E-MTAB-2298.

### Lipid mass spectrometry

Cell pellets were dissolved in 0.2% SDS solution. An aliquot corresponding to 100 μg was used for mass spectrometric lipid analysis. Lipid extraction was performed according to the method of Bligh and Dyer [21] in the presence of not naturally occurring lipid species as internal standards and the chloroform phase was dried in a vacuum centrifuge and dissolved in 10 mM ammonium acetate in methanol/chloroform (3∶1 vol/vol). Samples were analyzed by ESI-MS/MS in positive ion mode by direct flow injection using the analytical setup and data analysis algorithms described previously by Liebisch et al. [22]. PE-based plasmalogens were quantified according to the principles described by Zemski Berry and Murphy [18]. For this purpose fragment ions of m/z 364, 380 and 382 were used for PE P-16:0, PE P-18:1 and PE P-18:0 species, respectively. After identification of relevant lipid species, selected ion monitoring analysis was performed. Material from all thirteen donors was analyzed for differentiation days 1, 4 and 5. 0d monocytes were additionally available from four donors. Lipid nomenclature is used as recommended in [23].

### Statistical analysis

#### Partial least squares discrimination analysis (PLS/DA)

Partial least square discrimination analysis was performed and VIP values were calculated as described in [24]–[26]. The computation was performed using SPSS 20 (IBM) with the PLS extension module version 1.0.5 (IBM) under Python 2.7 with SciPy 0.11.0 (MLK) and NumPy 1.6.2 (MKL) on a 64 bit Windows 7 system (Microsoft).

#### Correlation analysis

Correlations between lipid species were calculated in SPSS 20 (IBM) using Pearson's product moment correlational analysis.

#### Evaluation of Agilent microarray data

Microarray experiments were analyzed according to the method described by Kondrakhin et al. [27]. With this method for each gene a so called hypergeometric score is calculated that considers the relative changes of an mRNA as well as the absolute signal intensity of the mRNA probe. Therefore relevant changes in gene expression can be identified more easily. Transcripts with calculated values <−4 or >+4 were considered significantly regulated. Gene Ontology enrichment analysis was performed on a ranked set of transcripts from microarray data with the BioUML software platform. For visualization only level 4 and level 5 categories were considered, primarily lipid related categories were chosen for visualization.

#### *In silico* transcription factor prediction

Significantly expressed and regulated lipid related genes were subjected to transcription factor and virtual promoter analysis, predicting potential transcriptional regulators. *In silico* promoter analysis was carried out using the TRANSFAC database and the ExPlain software system (BioBase, Germany). For in silico analysis we focused on regions of 1000 bp upstream from the transcription start site, and used a threshold in order to minimize false positive errors.

#### Partial correlation and trancriptional network visualization

q-order partial correlation graphs (qpgraphs) were used to reverse engineer the molecular regulatory network from microarray and lipid data. All data was Z-transformed prior to analysis and average non-rejection rates (NRR) were calculated in R 2.15.3 [28] using the package qpgraph [29]–[31]. The NRR ranges from 0 to 1 and helps in deciding what edges are present or missing from a qpgraph. In our experiments a threshold value of 0.3 was used for the non-rejection rate. The resulting network was visualized in the editor yEd 3.12 (yWorks, Germany).

## Results and Discussion

### Adaptions in the lipid related transcriptome enable monocytes for their phagocytic and inflammatory function

**Figure 1:**
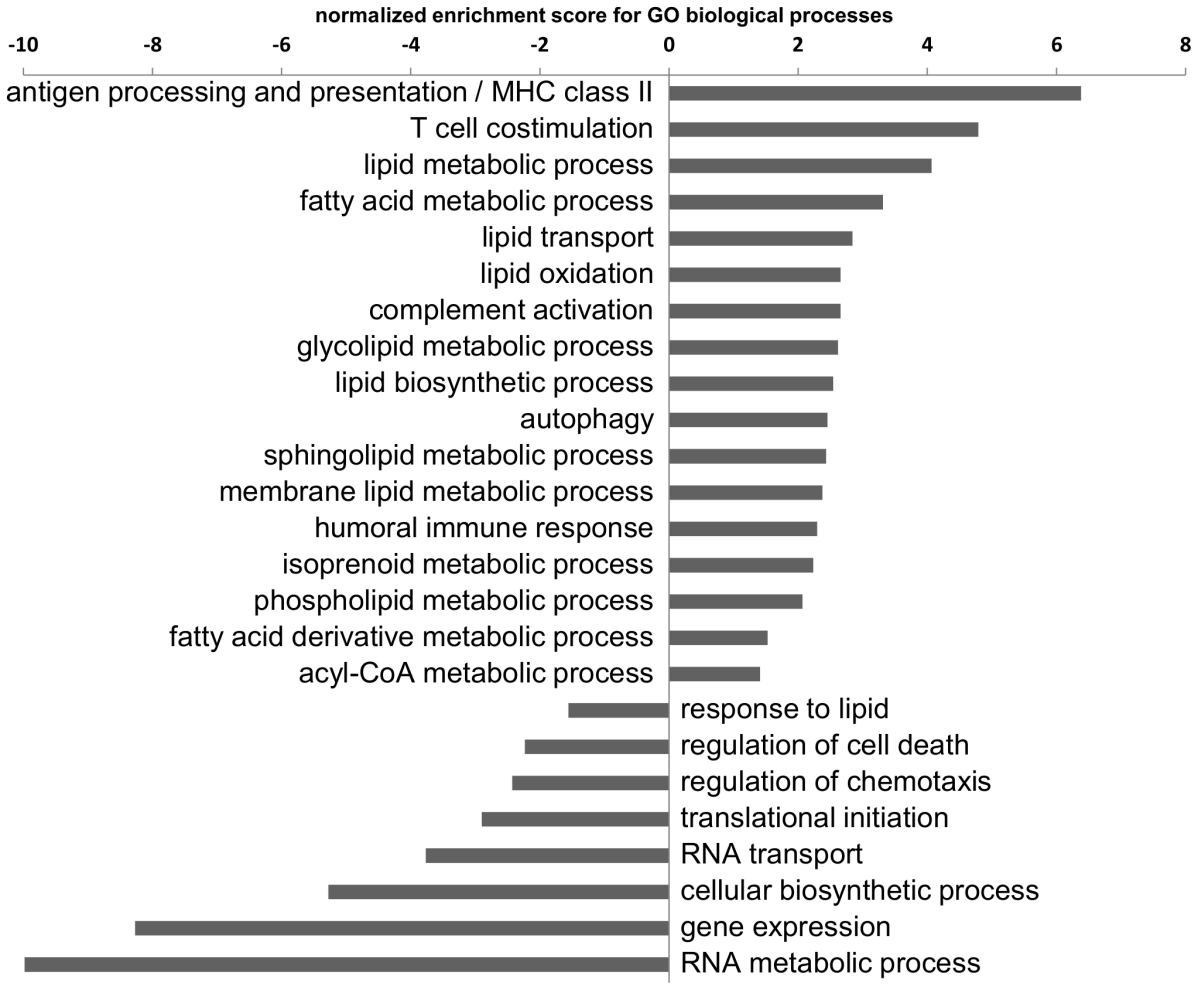
Gene ontology (GO) enrichment analysis of primary monocyte-macrophage differentiation. Normalized enrichment scores (NES) indicate the distribution of Gene Ontology categories across a list of genes ranked by hypergeometrical score (HGS). Higher enrichment scores indicate a shift of genes belonging to certain GO categories towards either end of the ranked list, representing up or down regulation (positive or negative values, respectively). Lipid related catergories are generally found to be shifted upwards.

Changes in the transcriptional profile during monocyte to macrophage differentiation were analyzed using microarrays for a comprehensive analysis of the cellular transcriptome at days 1, 4 and 5 of MCSF dependent *in vitro* differentiation. In total 4728 transcripts (34,1%) showed significant changes as indicated by a hypergeometrical score of <−4 or >+4 between days 1 and 4 of differentiation. Gene Ontology (biological process) analysis of the genearray data showed that lipid metabolism is among the most strongly positively enriched categories (Figure 1 and table S1). This is in line with our previously published results showing the importance of SREBP1/SP1 and SRF target genes in the differentiation process [17]. In the current study we therefore focused in depth on the regulation of genes involved in lipid metabolism, analyzed the lipidomic pattern by mass spectrometry and performed a bioinformatics analysis to study underlying regulatory mechanisms.

#### Fatty Acid Metabolism

**Figure 2:**
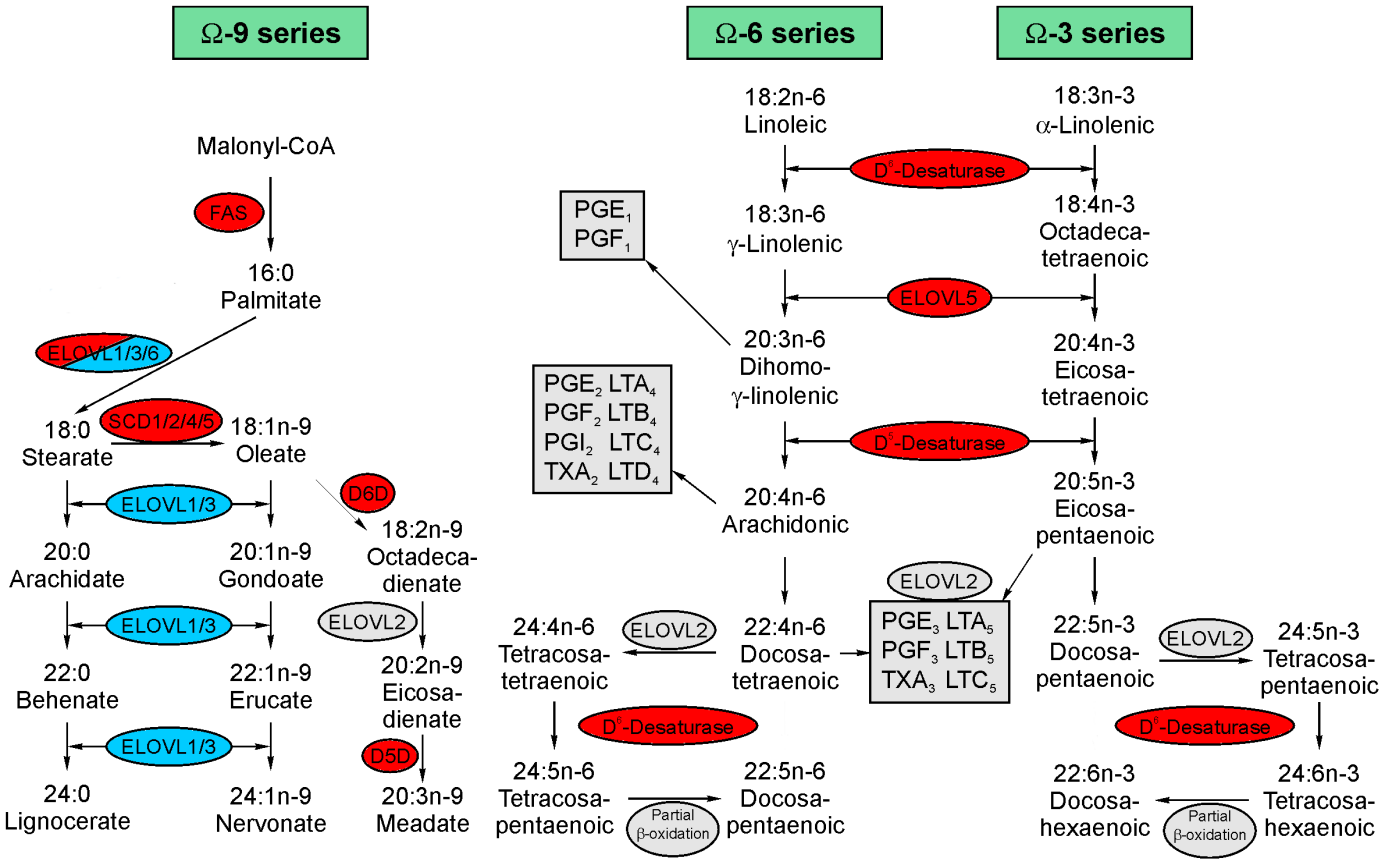
Schematic representation of fatty acid synthesis pathways. Fatty acid synthesis in the differentiation of primary human monocytes shows an induction of enzymes involved in the synthesis of palmitate (FAS), desaturation (SCD, D5D and D6D) and elongation (ELOVL5 & 6). Blue indicates significantly downregulated transcript levels, upregulated transcripts are shown in red and unchanged levels or unknown regulation in shown in grey.

Fatty acids are carboxylic acids with a long aliphatic tail. They are synthesized from acetyl-CoA and malonyl CoA precursors via six recurrent reactions that are catalyzed by the enzyme fatty acid synthase (FAS) until the 16-carbon product palmitic acid (C16:0) is produced. The main function of FAS is therefore to catalyze the synthesis of palmitate from acetyl-CoA and malonyl-CoA, in the presence of NADPH. The resulting saturated fatty acids are consecutively desaturated by desaturases or elongated by elongases at the endoplasmic-reticulum (ER). An overview over this process is given in Figure 2 with enzymes that were upregulated on the mRNA level shown in red and downregulated transcripts shown in blue. In our differentiation model transcripts for FAS were upregulated between days 1 and 4 and then remained stable at this level up to day 5, potentially leading to an increased synthesis of fatty acids. While the majority of cellular fatty acids that is synthesized via this pathway have a length of 16 to 18 carbon atoms, the cell also posseses the ability to elongate the carbon chain further using the ELOVL family of proteins. They catalyze the rate limiting condensation step in long chain fatty acid synthesis. ELOVL1 is ubiquitously expressed and catalyzes the condensation of very long chain (>C24) saturated or monounsaturated fatty acids [32]. During monocyte-macrophage differentiation, transcripts for ELOVL1 were downregulated. On the other hand transcripts for ELOVL5 and ELOVL6 were strongly induced. ELOVL5 processes a multitude of fatty acids including palmitoleic (C16:1, n-7), oleic (C18:1, n-9), γ-linolenic (C18:3, n-6), stearidonic (C18:4, n-3), arachidonic (C20:4, n-6) and eicosapentaenoic acid (EPA, C20:5, n-3) [33]. Adenoviral overexpression of ELOVL5 in primary rat hepatocytes has been reported to increase the elongation of arachidonic acid (20∶4,n-6) and eicosapentaenoic acid (20∶5,n-3) into adrenic acid (22∶4,n-6) and docosapentaenoic acid (DPA, 22∶5,n-3) respectively [34]. The monounsaturated fatty acids palmitic (16∶0, preferentially) and palmitoleic acid (16∶1) acid can be further elongated by ELOVL6 to stearic acid (18∶0) and oleic acid (18∶1) [35], [36]. During phagocytic differentiation transcripts for ELOVL6 were only transiently upregulated on day 4 and dropped again on day 5. Additionally ELOVL6 is responsible for the elongation of C12 to C16 saturated and monounsaturated long chain fatty acids [36]. Furthermore peroxisomal trans-2-enoyl-CoA reductase was also strongly induced and is known to play a role in chain elongation of fatty acids, as well as estradiol 17-beta-dehydrogenase 12, an enzyme that also has 3-ketoacyl-CoA reductase activity suggesting a role in long fatty acid elongation. Transcripts for 3-hydroxyacyl-CoA dehydratases 1 and 3 were upregulated. These enzymes are responsible for the dehydration step in very long-chain fatty acid (VLCFA) synthesis [37].

Desaturases introduce double bonds between defined carbon atoms in fatty acyl chains. In humans four types of fatty acid desaturases could be identified, according to the position of the double bond introduced. In our experiments all analyzed fatty acid desaturases were upregulated with the notable exception of FADS3, which is interesting since the gene encoding FADS3 is clustered with FADS1 and FADS2 [38]. The conversion of palmitic acid (16∶0) to palmitoleic acid (16∶1), as well as the conversion of stearic acid (18∶0) to oleic acid (18∶1) is mediated by delta-9-desaturase (D9D), also called stearoyl-CoA desaturase-1 (SCD1) or FADS5. In this study the expression of this rate-limiting microsomal enzyme was found to be strongly upregulated during phagocytic differentiation of monocytes. The monounsaturated fatty acids produced by SCD are further used as substrates in the production of cholesteryl esters, triglycerides, phospholipids and wax esters (reviewed in [39]). FADS1 (D5D), as well as FADS2 (D6D) are enzymes that are required for the synthesis of omega-3 and omega-6 long-chain polyunsaturated fatty acids. They therefore play a role in the synthesis of arachidonic acid as a precursor molecule for eicosanoid biosynthesis in omega-6 fatty acid synthesis and in the omega-3 line of EPA and DHA production. FADS1 is a delta-5-desaturase and FADS2 a delta-6-desaturase. Delta-4 desaturases primarily play a role in sphingolipid metabolism and were not significantly regulated by differentiation.

The mitochondrial very long-chain specific acyl-CoA dehydrogenase (ACADVL) was strongly up-regulated. Its function is the degradation of very long fatty acids by catalyzing the first step of the mitochondrial fatty acid beta-oxidation pathway. It can accommodate substrates with chain lengths as long as 24 C-atoms [40]. At the transcript level it was greatly diminished during differentiation, indicating a decreased degradation of these long chain fatty acids. On the other hand the dehydrogenase ACADSB that is preferentially active on short and branched chain acyl-CoAs was induced on days 4 and 5.

#### Lipoprotein Metabolism

Lipoproteins are aggregates of apo-lipoprotein (apo) and lipid that allow the transport of lipids in the aqueous blood stream. Especially plasmalogens are known to correlate positively to serum HDL levels and to decrease with aging [41]. During monocyte-macrophage differentiation apoE was strongly induced. ApoE is important for metabolizing triglyceride rich lipoproteins, is involved in vascular remodeling [42] and has been described to be antiatherogenic and to mediate cholesterol efflux. It also plays a role in the immune function of macrophages. Apolipoprotein J/clusterin is involved in cholesterol export from foam cells [43]. Its transcript levels were strongly increased after differentiation. Apolipoprotein C-I and C-II were also strongly upregulated. ApoC-I in plasma is mainly bound to VLDL, chylomicrons, and HDL [44]. In macrophages it has recently also been described to be able to bind lipopolysaccharide (LPS) [45]. ApoC-I regulates cholesterol and phospholipid efflux and seems to be crucially involved in the development of atherosclerotic lesions [46]. ApoC-II plays a role in the catabolism of triglyceride-rich lipoproteins and is also found on macrophages in atherosclerotic plaques [44]. It has the ability to activate lipoprotein-lipase (LPL). LPL is the primary enzyme for the hydrolysis of triacyl-glycerides in chylomicrons and VLDL. Its transcript was upregulated. Although LPL promotes foam cell formation and atherosclerosis [47], it is generally viewed as anti-atherogenic [48]. Macrophage LPL together with its activator protein apoC-II is thought to modulate macrophage inflammatory capacity through release of n-3/n-6 fatty acids. Their increased expression during differentiation reflects the increased inflammatory capacity of the macrophage by being able to access fatty acids for the synthesis of inflammatory mediators. Another enzyme potentially complementing LPL in the hydrolysis of monoglycerides is monoglyceride lipase (MGLL). It was found upregulated, facilitating hydrolysis of intracellular triglyceride stores to fatty acids and glycerol. The expression level of the transcript for the LDL receptor (LDLR) was only slightly diminished on day 4 and significantly downregulated on day 5, for the VLDL-receptor (VLDLR) we found no changes in expression.

Accumulation of excess free cholesterol in macrophages contributes to the development of atherosclerosis by stimulating tumor necrosis factor-α (TNF-α) and interleukin-6 production [49]. This leads to ER-stress and apoptosis [50]. In general enzymes involved in SREBP2 dependent cholesterol biosynthesis were downregulated while proteins involed in cholesterol export were strongly induced. The levels of the transcripts for the rate-limiting enzyme in cholesterol synthesis HMG-CoA reductase [51] were unchanged on day 4 and downregulated on day 5. Also transcript levels for squalene oxidase, as well as lathosterol oxidase were downregulated on both days. Cholesterol export from macrophages is largely mediated by the transporter ABCA1, but also to a smaller extent by ABCG1 [52]–[54]. It was found to be protective against cardiovascular disease and fulfill an anti-inflammatory function [52], [55], [56]. Interestingly this role seems to be independent of cholesterol transport. Its expression was strongly induced by differentiation. The cytosolic acetoacetyl-CoA thiolase (ACAT2), the main enzyme mediating esterification of cholesterol in the liver [57] was downregulated on day 5.

#### Plasmalogens, Glycerophospholipids and Eicosanoid Biosynthesis

**Figure 3:**
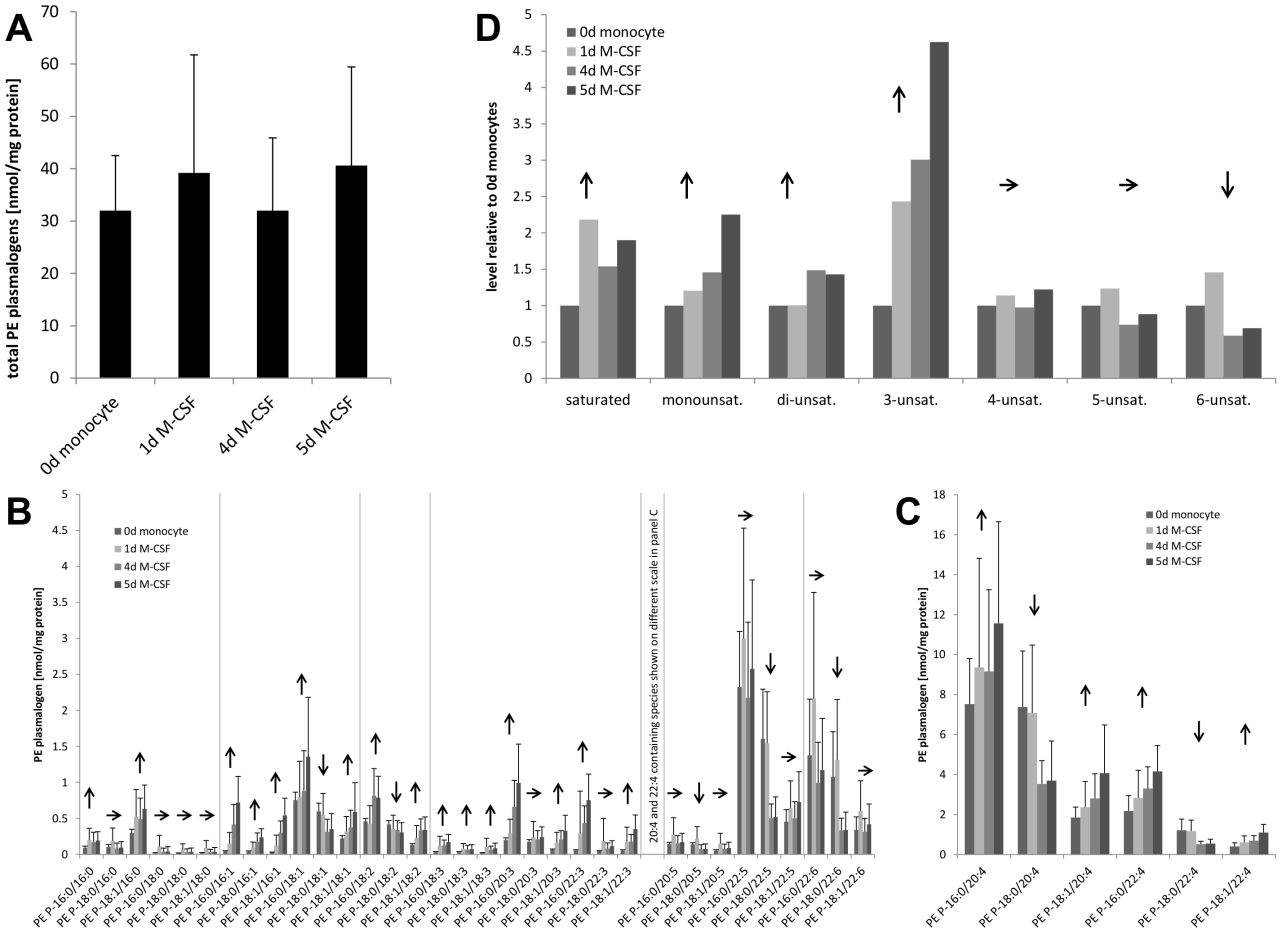
Regulation of PE plasmalogens during monocyte-macrophage differentiation. (A) Levels of total plasmalogens over thre course of 5 day primary monocyte macrophage differentiation. Individual PE plasmalogen species show specific changes during differentiation (B and C). Saturation specificity relative to day 0 is shown in panel (D).

Plasmalogens are ether glycerol-phospholipids that are integral parts of the plasma membrane as well as storage lipids for acyl residues and cellular anti-oxidants. Their synthesis takes place in a non-redundant pathway in peroxisomes and at the ER-membrane. Interestingly, although the macrophage is prepared for an immune reaction, transcripts for enzymes in plasmalogen synthesis remained mostly unchanged. The rate limiting enzyme for this process, glyceronephosphate O-acyltransferase (GNPAT, also called DHAP-AT), was not regulated on the transcriptomic level. The acyl residues in phosphatidyl-ethanolamine (PE) plasmalogens are taken from the cellular free fatty acid pool. For this purpose the enzyme fatty acyl-CoA reductase 1 (FAR1) provides the long chain fatty alcohol by reduction of saturated fatty acyl-CoAs in the peroxisomal membrane. During monocyte-macrophage differentiation its mRNA was downregulated. The resulting alcohols are then further processed by alkyldihydroxyacetonephosphate synthase (AGPS/ADHAP-S) in the peroxisome, an enzyme whose transcript was downregulated on day 4 and increased on day 5. These findings are in agreement with constant levels of total plasmalogens that were detected in lipid mass spectrometry (Figure 3A).

Eicosanoids are inflammatory mediators that are synthesized in macrophages from eicosapentaenoic acid (EPA, n-3), arachidonic acid (AA, n-6), and dihomo-gamma-linolenic acid (DGLA, n-6) [58], [59]. Plasmalogens in the plasma membrane represent a major reservoir for the storage of these precursors in mammalian cells (reviewed in [11]). Hydrolysis of the ester-bond in the sn-2 position is the first step in eicosanoid synthesis from precursor acyl residues in plasmalogens. Phospholipase A_2_ isoforms constitue a large family of enzymes specifically produced for this purpose. With the exception of PLA2G4A all PLA2 and PLD isoforms that were analyzed on the microarray showed either stable or increasing expression levels for their mRNA, while the endoperoxide synthases were downregulated. This points towards a tight regulation of eicosanoid production and processing. PLA2 is required for releasing precursor molecules for eicosanoid biosynthesis from either phosphatidylcholine (PC, esters) or plasmalogens (vinyl-ethers). Although plasmalogen specific PLA2 activity has been decribed in the literature, this enzymatic activity could not be associated with a specific isoform or transcript in the phospholipase family [60].

Eicosanoid production is subsequently mediated by the PG-endoperoxide synthases PTGS1 and PTGS2, also known as cyclooxigenases COX1 and COX2 [61]. Transcripts for both enzymes were downregulated, reaching significance for PTGS1 and showing a trend for PTGS2 in the hypergeometrical score. On the other hand arachidonate 5-lipoxygenase (ALOX5), and leukotriene A4 hydrolase (LTA4H) were strongly induced.

In summary these changes in the gene expression profile reflect the major alterations in lipid metabolism: increases in fatty acid synthesis, elongation and desaturation as well as increased cholesterol production, utilization and export from phagocytic macrophages in comparison to blood monocytes. Since the modulated release of inflammatory mediators is a key contribution of macrophages in the development and sustenance of atherosclerotic plaques we next focused on an in depth examination of plasmalogen lipidomics.

### The PE plasmalogen profile matures during monocyte macrophage differentiation

As already mentioned before plasmalogens regulate cell membrane fluidity and represent cellular stores for the precursor molecules of eicosanoid biosynthesis, mainly arachidonic acid [11]. Cellular PE-plasmalogen content was analyzed in monocytes and monocyte-derived macrophages at days 0, 1, 4 and 5 of an *in vitro* differentiation protocol. In general plasmalogen species containing AA in sn-2 position were most abundant. During the course of monocyte to macrophage differentiation, total plasmalogen levels did not change significantly (Figure 3A, p = 0.186, independent samples Kruskal-Wallis test due to varying number of experiments per time point). However, at the species level we found specific alterations in the plasmalogen species composition (Figure 3B and C). Especially minor plasmalogen species showed saturation dependent changes in concentration. As shown in Figure 3D, plasmalogen species containing poly-unsaturated (PUFA, five or six double bonds) or saturated acyl residues were typically downregulated while species with sn-2 bound residues with one, two or three double bonds were largely upregulated. Species containing AA (20∶4) did not show significant variations in their levels.

Clinically atherogenesis starts with increased deposition and modification of LDL in the subendothelium. Subsequently inflammatory cells such as monocytes are chemotactically attracted and migrate into the vessel wall. There they secrete reactive oxygen species (ROS) producing an environment with high levels of oxidative stress, that also affects the deposited LDL (reviewed in [62]). Interestingly LDL particles from obese metabolic syndrome patients and type 2 diabetics showed changes in their lipid composition that were similar to the changes we observed during monocyte-macrophage differentiation. Low density lipoprotein from obese subjects contained generally lower levels of PE plasmalogens and decreased proportions of PUFA in their cholesteryl esters, while saturated fatty acids (SFA) and mono-unsaturated fatty acids (MUFA) proportions were increased. Similarly LDL particles from type 2 diabetic patients contained decreased PUFA, had an unchanged MUFA content and showed increased SFA [63].

**Figure 4:**
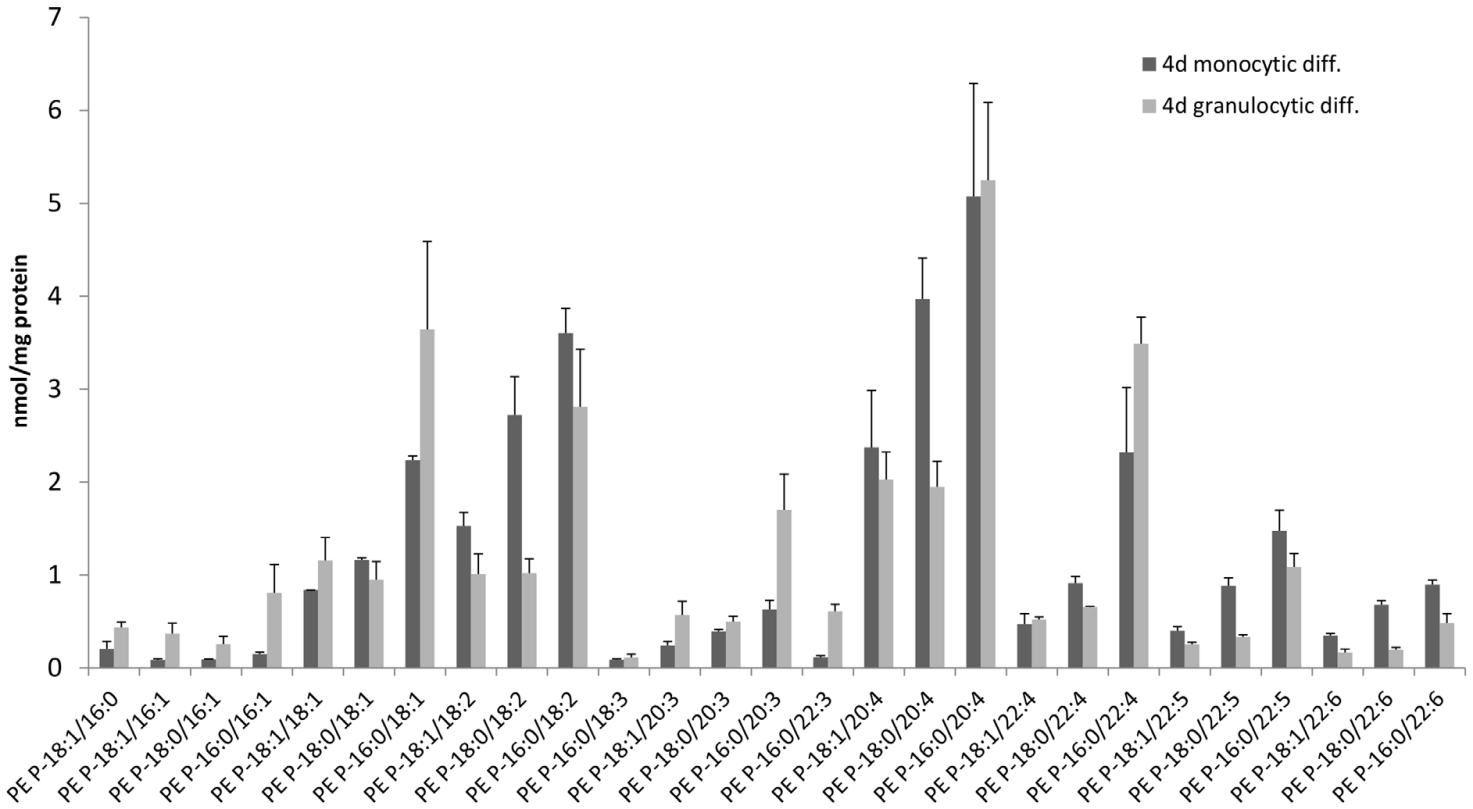
HL-60 model for monocyte and granulocyte differentiation. PE plasmalogen species in an *in vitro* model of monocytes and granulocytes using HL-60 cells show higher levels of species containing highly unsaturated acyl residues (>4 double bonds) in monocytic differentiation and higher levels of less unsaturated species (<4 double bonds), mono- or saturated species in granulocytic differentiation.

The PE plasmalogen pattern in human blood cells has been reported by our group before [12]. Interestingly the changes in the plasmalogen pattern during differentiation rendered a mature macrophage more similar to a blood granulocyte than the original monocyte. Since granulocytes represent fully mature cells from the same myeloid lineage as monocytes we suspected a general mode of regulation. In order to validate this pattern we used an *in vitro* model of HL-60 cells. Dependent on the stimulus, the pro-myelocytic cell line HL-60 can differentiate to a monocytic (Vit D_3_) as well as granulocytic (all-trans retinoic acid, ATRA) phenotype. We therefore analyzed the plasmalogen species pattern of differentiating HL-60 cells (Figure 4) and performed correlation analysis.

The correlation of HL-60 monocytic and granulocytic differentiation was strongly positive (Pearson r = 0.960, p<0.001). Furthermore we found a significant correlation between the changes in monocyte to macrophage differentiation and the regulation of plasmalogen species during monocytic differentiation of HL-60 cells (Pearson r = −0.522, p = 0.005). In contrast the changes found in HL-60 derived granulocytic cells were statistically different from monocyte-macrophage differentiation (Pearson r = −0.359, p = 0.066).

### Quantification of PE plasmalogen species allows discrimination of the differentiation state in a partial least squares discriminant analysis (PLS/DA)

Since the plasmalogen pattern undergoes very specific changes during phagocytic differentiation we hypothesized that it could serve as a biomarker for predicting the monocyte to macrophage differentiation state. To this end a PLS/DA analysis was performed on the data obtained from primary human monocytes and macrophages. PLS is a mathematical method that allows to analyze the influence of a matrix of predictors on an outcome variable.

**Figure 5:**
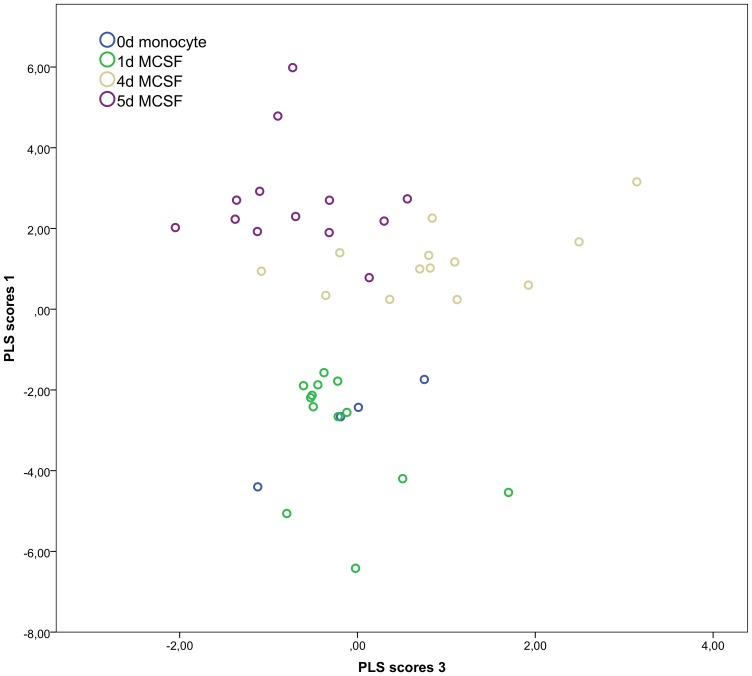
Partial least squares discriminant analysis (PLS/DA) during the course of primary monocyte-macrophage differentiation. While latent variable LV1 is able to discriminate between days 0/1 and days 4/5 of differentiation, LV4 can differentiate between days 4 and 5.

In this approach 4 different latent variables were computed and plotted graphically to evaluate their power to differentiate the differentiation stage. As shown in Figure 5, latent variable 1 allowed the clear discrimination of cells on days 0 and 1 from more mature cells on days 4 and 5. On the other hand latent variable 3 permitted to partially discern between days 4 and 5. A distinction between day 0 and day 1 was not possible using this approach. Therefore while there was no clear difference in the PE-plasmalogen pattern between undifferentiated monocytes and monocytes that had been treated with rhMCSF for 1d, cells that had been cultured with M-CSF for 4 or 5 days were clearly different in their lipidomic profile.

We also determined the most influential lipid species by calculating so called Variable Importance for the Projection (VIP) values as shown in Figure S1. The PE-plasmalogen species that showed the strongest potential to discriminate between monocytes and macrophages (mean PLS VIP 1) were minor species containing docosapentaenoic acid (DPA, 22∶5), docosahexaenoic acid (DHA, 22∶6) and palmitoleic acid (16∶1) esterified in sn-2 position. On the other hand linoleic acid (18∶2) containing species were best in discerning between days 4 and 5 of differentiation.

Chronic, systemic low-grade inflammatory diseases such as diabesity and the metabolic syndrome are characterized by a strong activation of tissue macrophages that chemotactically also attract additional monocytes from the circulating blood stream [64]. Therefore it is likely that such a chronic inflammatory reaction will also be reflected in the plasmalogen pattern of plasma samples or be detectable in isolated blood monocytes, rendering it a potential lipidomic biomarker.

### Transcriptional network analysis for the identification of novel PE plasmalogen related key nodes

**Figure 6:**
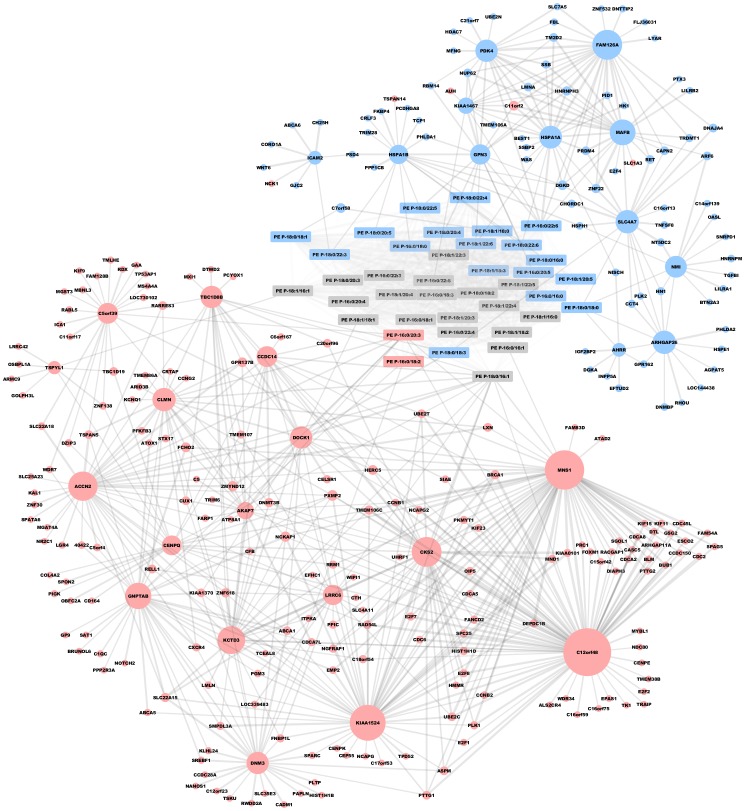
Molecular network graph of genes correlating to PE plasmalogens during primary monocyte-macropahge differentiation. Plasmalogens are shown in the center (rectangles) and correlating transcripts are grouped around them (circles). Circle size represents the number of connected nodes, line width the strength of the correlation and color the tyoe of regulation (upregulated in red, downregulated in blue). Central key nodes can be identified that have a multitude of connections to other nodes.

In order to identify novel players influencing the regulation of cellular plasmalogens we performed partial correlation analysis of plasmalogen species with gene array data (Figure 6). This bioinformatic approach allows to reduce the complexity of the model by eliminating indirect interactions and therefore to better identify key nodes. Among the transcripts found to be central by this algorithm were well known factors proving the plausibility of the method. For example MAFB is a known inducer of monocytic differentiation [65] that showed up centrally. Also the 70 kDa heatshock proteins HSPA1B and HSPA1A were central nodes. Hsp70 proteins act as molecular chaperones that can maintain the survival of stressed cells [66]–[69]. They accomplish this by stabilizing lysosomal membranes through the binding of BMP [70]. For these proteins genotypic variations have been described that play a role in cytokine release [71].

Furthermore a range of proteins that have not yet been implicated in monocyte-macrophage differentiation were identified. DOCK1 is a Rac activator that regulates myoblast fusion [72]. It is involved in regulating cell surface extensions and has the ability to bind phospholipids via a DHR1 domain [73]. Thereby it posseses the ability to interact with cell membranes, a function that is essential for differentiated macrophages. In our cells its transcript levels were significantly upregulated during the differention process. Pyruvate dehydrogenase lipoamide kinase isozyme 4 (PDK4) is a mitochondrial enzyme regulating glucose metabolism and therefore cellular energy homeostasis. Strongly decreased expression of PDK4 as found in our analysis increases metabolism and especially conversion of glucose to acetyl-CoA, thereby improving substrate availability for fatty acid synthesis. Moreover N-acetylglucosamine-1-phosphate transferase (GNPTAB, significantly upregulated during differentiation) is involved in synthesis of mannose-6-phosphate, that itself tags hydrolases for transfer to the lysosome. The expression of this enzyme could therefore have influence on the protein composition of the macrophage lysosomes. The protein encoded by FAM126A may play a part in the beta-catenin/Lef signaling pathway and has not been described to play a role in macrophages before. In our cells its mRNA levels were downregulated during differentiation. C12orf48 enhances PARP-1 activity, protecting from DNA damage [74], knockdown was anti-proliferative in HeLa cells [75]. It was upregulated. Meiosis-specific nuclear structural 1 (MNS1, significantly upregulated) has only been described to play a role in the regulation of meiosis before [76]. Its function in monocytes and macrophages has not been analyzed yet.

Further transcripts that were identified include ARHGAP26, a Rho GTPase involved in the formation of endocytotic vesicles, cell spreding and adhesion [77], ACCN2, a sodium channel [78] and KIAA1524, a phosphatase inhibitor that plays a role in oncogenesis [79].

Further studies are needed to clarify the role of these potential lipid related modulators of the differentiation process.

### Identifying underlying transcription factors

Major regulatory changes in the cellular transcriptional profile are mediated by transcription factors. We used a bioinformatics approach therefore affecting all target genes that contain a specific binding motif in their promoter region at the same time. This search was based on transcripts listed in the Gene Ontology category lipid metabolism. Transcription factors that are predicted to bind to promoters of these genes are shown in Table 1. On the one hand this approach predicted well known factors essential in macrophage differentiation such as PPARG, KLF4 [80] and EGR1 [81], [82] proving the feasibility of the algorithm. The binding motifs identified in this approach also allow binding of previously described factors that regulate lipid associated genes such as SREBP1, although it does not show up in the analysis directly. SREBP1 is known to interact with Sp1 at Sp1/Sp4 sites in regulating gene expression, as well as with Nuclear factor Y (NFY). Therefore combinations of these factors determine the regulation of distinct cellular pathways [83]. SREBP1 target genes include fatty acid synthase and the LDL-receptor. Transcript levels for both were strongly induced during the differentiation process. SREBP1 also posses the ability to cooperate with CREB-binding protein as a co-activator. This combinatorial regulation of multiple transcription factors is also likely to be the reason for the occurrence of the motifs for EGR and p53 in up- and downregulated genes at the same time. On the other hand an involvement of previously not published binding motives could be predicted for the first time. These include ZNF148, nuclear factor Y and RNF96. Since transcription factor binding motif analysis was based solely on lipid metabolism related transcripts, it is likely that some of the known effects of these transcription-factors on differentiation are mediated in turn by lipidomic changes in the cell.

**Table 1:** Results of an in silico transcription factor binding motif analysis.

Up-regulated genes	Down-regulated genes
GZF1	CHCH
LXR	PPARG
TATA	HIF2A
IRF	ZBTB
ZNF148	ELF ETS
ATF CREB	AP1
TFE	ZFX
GKLF	FKLF
USF	CNOT3
FOX	RNF96
	MYCMAX
	AHRARNTHIF
	E2F

Up- and down-regulated genes
AP2
NFY
P53
SP1SP4
EGR

Motives that were enriched in the 1000 bp promoter region upstream of up-regulated lipid related genes are shown on the left, binding motives that were enriched in promoters of down regulated genes on the right and motifs that were associated with up- and down-regulated genes at the bottom. Abbreviations (as used in the Transfac database): AHRARNTHIF: aryl hydrocarbon receptor/aryl hydrocarbon receptor nuclear translocator/hypoxia inducible factor, AP1: activator protein 1, AP2: activating protein 2, ATF CREB: activating transcription factor/cAMP response element binding, CHCH: Churchill, CNOT3: CCR4-NOT transcription complex subunit 3, E2F: E2F transcription factor, EGR: early growth response, ELF ETS: E74-like factor/E26 transformation-specific, FKLF: fetal β-like globin gene-activating Krüppel-like factor, FOX: forkhead box, GKLF: gut-enriched Krüppel-like factor, GZF1: GDNF-inducible zinc finger protein 1, HIF2A: hypoxia-inducible factor 2a, IRF: interferon regulatory factor, LXR: liver X receptor, MYCMAX: Myc/Max transcriptional complex, NFY: nuclear factor Y, p53: phosphoprotein p53, PPARG: peroxisome proliferator-activated receptor-gamma, RNF96: RING finger protein 96, SP1SP4: specificity protein 1 and 4, TATA: TATA box, TFE: Transcription Factor E (Family), USF: upstream transcription factor, ZBTB: zinc finger and BTB (broad complex, tramtrack, and bric-à-brac) domain-containing, ZFX: zinc finger X-chromosomal protein, ZNF148: zinc finger protein 148.

## Summary and Conclusion

During the differentiation process to mature macrophages monocytes undergo profound changes in their lipidomic and transcriptomic profile to prepare them for their phagocytic and inflammatory function.

Fatty acid synthesis pathways show a strong induction of transcripts involved in fatty acid desaturation and elongation. Lipid transport and lipoprotein related transcripts are differentially modulated partly in a pro-inflammatory way partly anti-inflammatory. On the one hand cholesterol biosynthesis is downregulated and export is strongly promoted. At the same time the cell prepares to facilitate the hydrolysis of triglycerides. In depth analysis was perfomed for plasmalogens. Plasmalogen biosynthesis pathways remained mostly unchanged on the mRNA level. This was reflected in lipidomic measurements showing constant total plasmalogen levels. Plasmalogens represent storage lipids for precursor acyl-residues in eicosanoid synthesis. Transcripts for the iso-enzymes involved in eicosanoid production were tightly regulated.

Lipidomic analysis of PE plasmalogens showed decreases in polyunsaturated species (PUFA) and increases in monounsaturated species (MUFA). These adaptions rendered monocyte derived macrophages more similar to mature granulocytes in their plasmalogen profile. This observation could be confirmed in an HL-60 model system for granulocytic and monocytic *in vitro* differentiation. The PE plasmalogen species pattern could be shown to be specific for monocytes and monocyte derived macrophages permitting a purely lipidomic discrimination of the differention stage. It might therefore also represent a potential biomarker indicating immune system activation, e.g. in patients with metabolic syndrome that suffer from chronic, low-grade inflammation.

A bioinformatic approach using partial correlation and molecular network reconstruction was able to identify novel potential hubs in the plasmalogen related transcriptional network, such as 70 kDa heatshock proteins, DOCK1, PDK4, GNPTAB, FAM126A, MNS1 and C12orf48. Further studies are currently underway to clarify the potential modulatory role of these proteins during differentiation. Transcription factor motif analysis revealed the involvement of several binding motifs with the remarkable involvement of motifs for NFY, P53, SP1 and EGR in up- and down- regulated genes. Therefore probably the interplay of multiple additional factors such as SREBP, determines the amount of transcription of lipid regulatory transcripts.

In summary we could for the first time show the specificity of the changes in the plasmalogen pattern during monocyte to macrophage differentiation. Furthermore using a bioinformatics approach we were able to predict novel regulatory hubs and transcription factors that might play central roles in the complex interplay of transcription-factors, transcripts and lipids during monocyte-macrophage differentiation.

## Supporting Information

Figure S1 **Variable importance in projection for PE plasmalogens during differentiation.** The VIP value summarizes the contribution a variable makes to the model. Therefore higher values (especially values larger than 0.8) signify a more significant contribution of the respective lipid species to the estimation of the differentiation stage.
(TIF)

Table S1 **GO classification of enriched transcripts during differentiation**. Normalized enrichment scores (NES) were calculated to reflect the distribution of Gene Ontology categories across a list of genes ranked by hypergeometrical score (HGS). A higher enrichment score indicates a shift of genes belonging to certain GO categories towards either end of the ranked list, representing up or down regulation (positive or negative values, respectively).
(XLSX)

Table S2 **Hypergeometrical score of lipid related transcripts during differentiation**. Agilent microarray data shows regulatory activity in fatty acid desaturation and elongation as well as changes in plasmalogen synthesis and degradation. Comparisons were made for day 4 versus day 1 (d4) and for day 5 versus day 1 (d5). Blue color indicates a significantly lower value following differentiation while red indicates significantly higher values.
(DOCX)
