# Supplementary figures and images for: Monocyte to Macrophage Differentiation Goes along with Modulation of the Plasmalogen Pattern through Transcriptional Regulation
Source: PLoS One. 2014 Apr 8;9(4):e94102. doi: 10.1371/journal.pone.0094102 (PMC3979739; doi:10.1371/journal.pone.0094102)

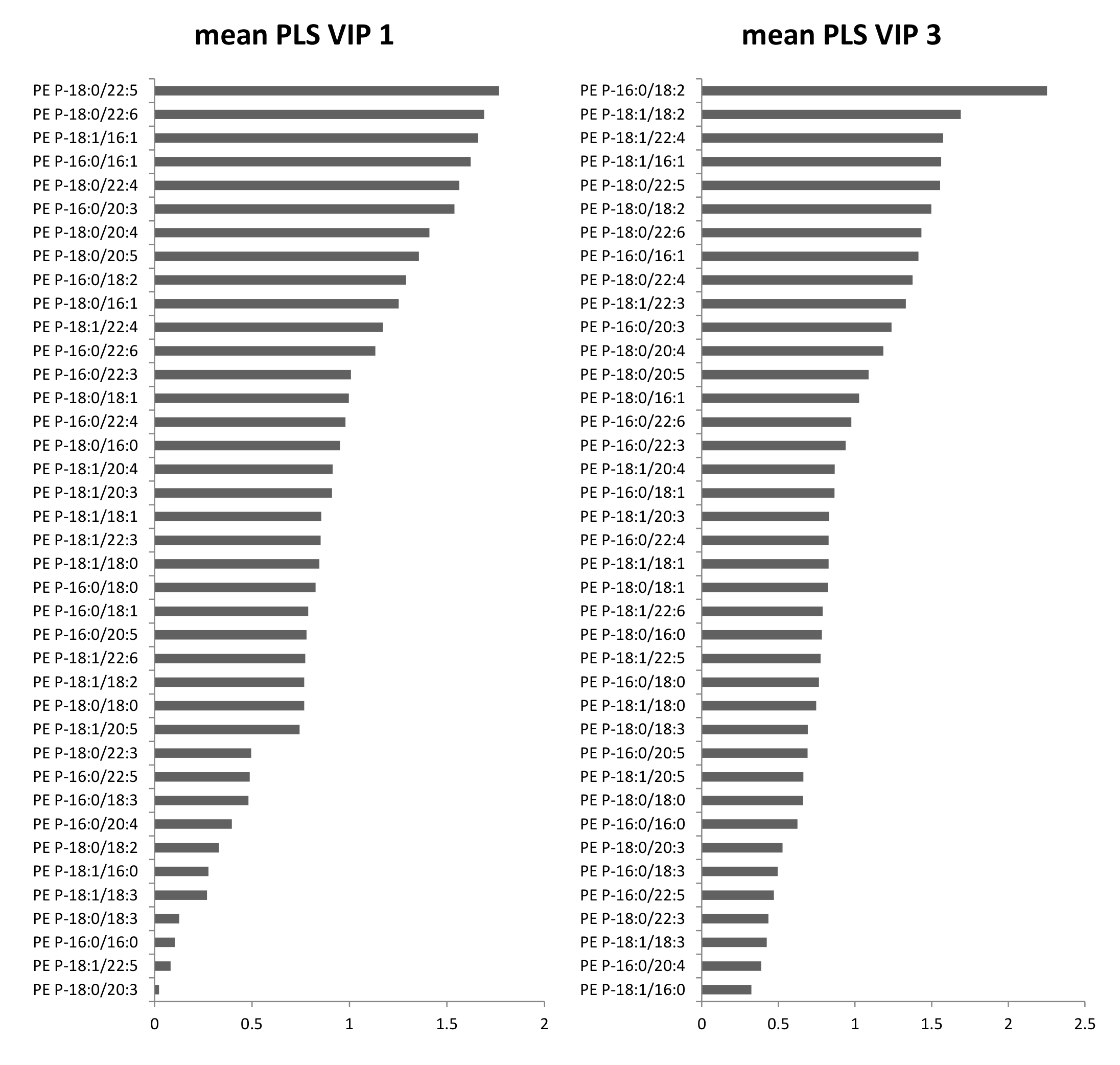

Supplement: Figure S1 — Variable importance in projection for PE plasmalogens during differentiation. The VIP value summarizes the contribution a variable makes to the model. Therefore higher values (especially values larger than 0.8) signify a more significant contribution of the respective lipid species to the estimation of the differentiation stage. (TIF) [file pone.0094102.s001.tif]
