# Supplementary material for: Monocyte to Macrophage Differentiation Goes along with Modulation of the Plasmalogen Pattern through Transcriptional Regulation
Source: PLoS One. 2014 Apr 8;9(4):e94102. doi: 10.1371/journal.pone.0094102 (PMC3979739; doi:10.1371/journal.pone.0094102)
Supplement: Table S2 — Hypergeometrical score of lipid related transcripts during differentiation. Agilent microarray data shows regulatory activity in fatty acid desaturation and elongation as well as changes in plasmalogen synthesis and degradation. Comparisons were made for day 4 versus day 1 (d4) and for day 5 versus day 1 (d5). Blue color indicates a significantly lower value following differentiation while red indicates significantly higher values. (DOCX) [file pone.0094102.s003.docx]

Table S2: mRNA array data show regulatory activity in fatty acid desaturation and elongation, as well as changes in plasmalogens synthesis and degradation

| **symbol** | **description** | | **d4** | **d5** |
| --- | --- | --- | --- | --- |
| **Fatty acid metabolism** | |  |  |  |
| AASDHPPT | L-aminoadipate-semialdehyde dehydrogenase-phosphopantetheinyl transferase | | -5,1 | -5,3 |
| ACACA | Acetyl-CoA carboxylase 1 | | -4,5 | -7,4 |
| ACACB | Acetyl-CoA carboxylase 2 | | 4,7 | 4,9 |
| ACADVL | Very long-chain specific acyl-CoA dehydrogenase, mitochondrial | | -9,2 | -10,3 |
| ACSF3 | Acyl-CoA synthetase family member 3, mitochondrial | | 3,8 | 5,7 |
| ACSL3 | Long-chain-fatty-acid--CoA ligase 3 | | 6,5 | 5,2 |
| ACSM3 | Acyl-coenzyme A synthetase ACSM3, mitochondrial | | 3,0 | 1,8 |
| APOC1 | Apolipoprotein C-I | | 17,8 | 18,0 |
| APOC2 | Apolipoprotein C-II | | 13,3 | 14,6 |
| BRCA1 | Breast cancer type 1 susceptibility protein | | 12,8 | 11,0 |
| C5orf4 | Uncharacterized protein C5orf4 | | 14,3 | 14,1 |
| CBR4 | Carbonyl reductase family member 4 | | 5,1 | 2,5 |
| CH25H | Cholesterol 25-hydroxylase | | -15,9 | -8,2 |
| DECR2 | Peroxisomal 2,4-dienoyl-CoA reductase | | -1,2 | -1,9 |
| DEGS1 | Sphingolipid delta(4)-desaturase DES1 | | 1,3 | 2,0 |
| DEGS2 | Sphingolipid delta(4)-desaturase/C4-hydroxylase DES2 | | -1,4 | -1,7 |
| ELOVL1 | Elongation of very long chain fatty acids protein 1 | | -5,8 | -6,7 |
| ELOVL5 | Elongation of very long chain fatty acids protein 5 | | 8,7 | 7,7 |
| ELOVL6 | Elongation of very long chain fatty acids protein 6 | | 6,5 | 2,9 |
| FA2H | Fatty acid 2-hydroxylase | | 8,2 | 9,8 |
| FADS1 | Fatty acid desaturase 1 | | 7,4 | 4,6 |
| FADS2 | Fatty acid desaturase 2 | | 7,3 | 5,8 |
| FADS3 | Fatty acid desaturase 3 | | -10,7 | -11,4 |
| FASN | Fatty acid synthase | | 4,4 | -1,0 |
| HNF1A | Hepatocyte nuclear factor 1-alpha | | 0,9 | 0,4 |
| HSD17B12 | Estradiol 17-beta-dehydrogenase 12 | | 5,0 | 2,0 |
| HSD17B8 | Estradiol 17-beta-dehydrogenase 8 | | 1,1 | 1,3 |
| INSIG1 | Insulin-induced gene 1 protein | | 1,7 | -3,5 |
| INSIG2 | Insulin-induced gene 2 protein | | 1,4 | 2,9 |
| LPL | Lipoprotein lipase | | 8,1 | 6,2 |
| MCAT | Malonyl-CoA-acyl carrier protein transacylase, mitochondrial | | -2,2 | -3,4 |
| MECR | Trans-2-enoyl-CoA reductase, mitochondrial | | -2,3 | -2,7 |
| MGLL | Monoglyceride lipase | | 6,2 | 7,1 |
| MID1IP1 | Mid1-interacting protein 1 | | 6,6 | 7,4 |
| MLXIPL | Carbohydrate-responsive element-binding protein | | 16,0 | 11,2 |
| MLYCD | Malonyl-CoA decarboxylase, mitochondrial | | -1,4 | -3,0 |
| MYO5A | Unconventional myosin-Va | | 5,0 | 4,5 |
| NDUFAB1 | Acyl carrier protein, mitochondrial | | -1,7 | -2,1 |
| NR1H2 | Oxysterols receptor LXR-beta | | -1,0 | 3,1 |
| NR1H3 | Oxysterols receptor LXR-alpha | | 17,7 | 17,6 |
| OXSM | 3-oxoacyl-[acyl-carrier-protein] synthase, mitochondrial | | -1,4 | -3,0 |
| PCCB | Propionyl-CoA carboxylase beta chain, mitochondrial | | 7,7 | 4,9 |
| PECR | Peroxisomal trans-2-enoyl-CoA reductase | | 8,5 | 8,4 |
| PRKAA1 | c-AMP-activated protein kinase catalytic subunit alpha-1 | | 2,0 | 2,1 |
| PRKAB1 | c-AMP-activated protein kinase subunit beta-1 | | -3,2 | -4,2 |
| PRKAB2 | c-AMP-activated protein kinase subunit beta-2 | | -2,7 | -0,9 |
| PRKAG1 | c-AMP-activated protein kinase subunit gamma-1 | | -0,6 | -1,1 |
| PRKAG2 | c-AMP-activated protein kinase subunit gamma-2 | | -2,2 | 1,5 |
| PTPLA | 3-hydroxyacyl-CoA dehydratase 1 | | 15,9 | 12,5 |
| PTPLAD1 | 3-hydroxyacyl-CoA dehydratase 3 | | 7,0 | 6,0 |
| PTPLAD2 | 3-hydroxyacyl-CoA dehydratase 4 | | 1,9 | 2,7 |
| PTPLB | 3-hydroxyacyl-CoA dehydratase 2 | | -2,3 | -2,9 |
| QKI | Protein quaking | | -5,1 | 3,2 |
| SC5DL | Lathosterol oxidase | | -6,2 | -6,3 |
| SCAP | Sterol regulatory element-binding protein cleavage-activating protein | | -4,7 | -5,4 |
| SCD | Acyl-CoA desaturase | | 16,7 | 14,0 |
| SCD5 | Stearoyl-CoA desaturase 5 | | 3,3 | 2,9 |
| STK11 | Serine/threonine-protein kinase STK11 | | -4,8 | -3,7 |
| TECR | Trans-2,3-enoyl-CoA reductase | | 3,2 | 2,1 |
| TRIB3 | Tribbles homolog 3 | | 2,1 | 3,8 |
| WDTC1 | WD and tetratricopeptide repeats protein 1 | | 1,6 | 2,6 |
| **plasmalogen metabolism** | |  |  |  |
| AGPS | Alkylglycerone phosphate synthase | | -4,5 | 5,1 |
| FAR1 | Fatty acyl-CoA reductase 1 | | -7,8 | -7,0 |
| FAR2 | Fatty acyl-CoA reductase 2 | | -1,3 | -2,1 |
| GNPAT | Dihydroxyacetone phosphate acyltransferase | | 0,9 | -0,7 |
| PEX7 | Peroxisomal targeting signal 2 receptor | | 1,9 | 1,0 |
| **phospholipid biosynthetic process** | |  |  |  |
| AGPAT3 | 1-acyl-sn-glycerol-3-phosphate acyltransferase gamma | | -3,4 | -5,4 |
| AGPAT4 | 1-acyl-sn-glycerol-3-phosphate acyltransferase delta | | 10,4 | 8,5 |
| AGPAT5 | 1-acyl-sn-glycerol-3-phosphate acyltransferase epsilon | | -8,2 | -9,4 |
| CDIPT | CDP-diacylglycerol--inositol 3-phosphatidyltransferase | | 5,9 | 5,9 |
| CDS2 | Phosphatidate cytidylyltransferase 2 | | 10,1 | 10,4 |
| CHPT1 | Cholinephosphotransferase 1 | | 9,4 | 10,1 |
| CRLS1 | Chromosome 20 open reading frame 155, isoform CRA_b | | -0,8 | -2,7 |
| DGKE | Diacylglycerol kinase epsilon | | -2,4 | 1,0 |
| FADS1 | Fatty acid desaturase 1 | | 7,4 | 4,6 |
| GNPAT | Dihydroxyacetone phosphate acyltransferase | | 0,9 | -0,7 |
| GPAT2 | Glycerol-3-phosphate acyltransferase 2, mitochondrial | | 10,0 | 7,6 |
| HEXB | Beta-hexosaminidase subunit beta | | 8,2 | 9,2 |
| ISYNA1 | Inositol-3-phosphate synthase 1 | | 2,1 | -2,4 |
| LPCAT1 | Lysophosphatidylcholine acyltransferase 1 | | 1,1 | 1,1 |
| LPCAT3 | Lysophospholipid acyltransferase 5 | | 8,1 | 6,9 |
| LPCAT4 | Lysophospholipid acyltransferase LPCAT4 | | 2,8 | 2,9 |
| LPGAT1 | Acyl-CoA:lysophosphatidylglycerol acyltransferase 1 | | 2,6 | 1,5 |
| MBOAT1 | Lysophospholipid acyltransferase 1 | | 4,4 | 5,4 |
| MBOAT2 | Lysophospholipid acyltransferase 2 | | 2,5 | 1,3 |
| MBOAT7 | Lysophospholipid acyltransferase 7 | | -2,5 | -1,8 |
| PCYT2 | Ethanolamine-phosphate cytidylyltransferase | | 2,7 | -0,6 |
| PGS1 | cDNA FLJ55337, highly similar to Homo sapiens phosphatidylglycerophosphate synthase 1 (PGS1), mRNA | | -2,7 | -3,8 |
| PIP5K1A | Phosphatidylinositol 4-phosphate 5-kinase type-1 alpha | | -1,7 | -2,2 |
| PISD | Phosphatidylserine decarboxylase | | -2,0 | -3,5 |
| PLA2G16 | Group XVI phospholipase A1/A2 | | 5,3 | 4,5 |
| SERINC1 | Serine incorporator 1 | | 1,9 | 3,9 |
| SERINC5 | Serine incorporator 5 | | 4,1 | 5,1 |
| **phospholipases** | |  |  |  |
| PLA2G4A | phospholipase A2, group IVA (cytosolic, calcium-dependent) | | -4,4 | -5,2 |
| PLA2G4C | phospholipase A2, group IVC (cytosolic, calcium-independent) | | 3,4 | 5,9 |
| PLA2G4D | phospholipase A2, group IVD (cytosolic) | | -3,1 | -2,5 |
| PLA2G6 | phospholipase A2, group VI (cytosolic, calcium-independent) | | -0,3 | 1,1 |
| PLA2G7 | phospholipase A2, group VII (platelet-activating factor acetylhydrolase, plasma) | | 7,6 | 6,8 |
| PLA2G12A | phospholipase A2, group XIIA | | 9,2 | 8,7 |
| PLA2G15 | phospholipase A2, group XV | | 6,6 | 6,1 |
| PLA2G16 | phospholipase A2, group XVI | | 5,3 | 4,5 |
| PLD1 | phospholipase D1, phosphatidylcholine-specific | | 11,6 | 11,2 |
| PLD2 | phospholipase D2 | | -4,2 | -4,3 |
| PLD3 | phospholipase D family, member 3 | | 10,5 | 11,5 |
| PLD4 | phospholipase D family, member 4 | | 2,4 | 1,1 |
| PLD6 | phospholipase D family, member 6 | | -3,3 | -3,6 |
| **phosphatidylethanolamine biosynthetic process** | |  |  |  |
| CEPT1 | Choline/ethanolaminephosphotransferase 1 | | -3,7 | -3,6 |
| CHKA | Choline kinase alpha | | 3,6 | 3,8 |
| CHKB | Choline/ethanolamine kinase | | -4,0 | -5,0 |
| ETNK1 | Ethanolamine kinase 1 | | 3,2 | -2,7 |
| ETNK2 | cDNA FLJ52473, highly similar to Ethanolamine kinase 2 (EC 2.7.1.82) | | 4,2 | 2,9 |
| PCYT2 | Ethanolamine-phosphate cytidylyltransferase | | 2,7 | -0,6 |
| PISD | Phosphatidylserine decarboxylase proenzyme | | -2,0 | -3,5 |
| SLC27A1 | Long-chain fatty acid transport protein 1 | | 6,8 | 8,1 |
| AARS | Alanine--tRNA ligase, cytoplasmic | | -2,2 | 1,5 |
| ACADVL | Very long-chain specific acyl-CoA dehydrogenase, mitochondrial | | -9,2 | -10,3 |
| ADD1 | Alpha-adducin | | -2,0 | 3,4 |
| AIFM1 | Apoptosis-inducing factor 1, mitochondrial | | 5,4 | 3,3 |
| AMFR | E3 ubiquitin-protein ligase AMFR | | 9,0 | 9,8 |
| ARFGAP1 | ADP-ribosylation factor GTPase-activating protein 1 | | -2,8 | -3,3 |
| ASNS | Asparagine synthetase [glutamine-hydrolyzing] | | -7,6 | -6,6 |
| ATF3 | Cyclic AMP-dependent transcription factor ATF-3 | | 3,5 | 1,3 |
| ATF4 | Cyclic AMP-dependent transcription factor ATF-4 | | -2,7 | 2,2 |
| ATF6 | Cyclic AMP-dependent transcription factor ATF-6 alpha | | -1,7 | -1,0 |
| ATG10 | Ubiquitin-like-conjugating enzyme ATG10 | | 3,1 | 1,8 |
| ATP6V0D1 | V-type proton ATPase subunit d 1 | | 2,6 | 2,0 |
| BAK1 | Bcl-2 homologous antagonist/killer | | 1,4 | -0,8 |
| BAX | Apoptosis regulator BAX | | -2,8 | -2,7 |
| BCL2 | Apoptosis regulator Bcl-2 | | 2,2 | 3,0 |
| C19orf10 | UPF0556 protein C19orf10 | | 1,8 | 0,9 |
| CALR | Calreticulin | | -2,7 | -5,5 |
| CCDC47 | Coiled-coil domain-containing protein 47 | | -1,2 | -1,7 |
| CCL2 | C-C motif chemokine 2 | | -2,5 | -3,2 |
| CCND1 | G1/S-specific cyclin-D1 | | 17,0 | 12,9 |
| CHAC1 | Cation transport regulator-like protein 1 | | -4,0 | -2,1 |
| COL4A3BP | Collagen type IV alpha-3-binding protein | | 9,3 | 9,4 |
| CREB3 | Cyclic AMP-responsive element-binding protein 3 | | 3,1 | 2,6 |
| CREB3L2 | Cyclic AMP-responsive element-binding protein 3-like protein 2 | | 11,3 | 10,5 |
| CTDSP2 | Carboxy-terminal domain RNA polymerase II polypeptide A small phosphatase 2 | | 1,0 | 2,7 |
| CTH | Cystathionine gamma-lyase | | 10,0 | 9,5 |
| CUL7 | Cullin-7 | | 5,5 | 5,8 |
| CXXC1 | CpG-binding protein | | -5,5 | -4,8 |
| DCTN1 | Dynactin subunit 1 | | -1,1 | -0,3 |
| DDIT3 | DNA damage-inducible transcript 3 protein | | -3,8 | -3,7 |
| DDX11 | Probable ATP-dependent RNA helicase DDX11 | | 3,4 | 0,8 |
| DERL1 | Derlin-1 | | -1,7 | -2,7 |
| DERL2 | Derlin-2 | | -4,4 | -3,7 |
| DERL3 | Derlin-3 | | 1,4 | -2,4 |
| DNAJB11 | DnaJ homolog subfamily B member 11 | | -5,7 | -7,0 |
| DNAJB9 | DnaJ homolog subfamily B member 9 | | 1,2 | 1,7 |
| DNAJC10 | DnaJ homolog subfamily C member 10 | | -2,4 | -2,4 |
| DNAJC3 | DnaJ homolog subfamily C member 3 | | -1,7 | -2,2 |
| EDEM1 | ER degradation-enhancing alpha-mannosidase-like 1 | | -2,4 | -3,7 |
| EIF2AK2 | Interferon-induced, double-stranded RNA-activated protein kinase | | -1,2 | 0,7 |
| EIF2AK3 | Eukaryotic translation initiation factor 2-alpha kinase 3 | | -1,6 | -0,8 |
| EIF2AK4 | Eukaryotic translation initiation factor 2-alpha kinase 4 | | 3,1 | 4,9 |
| EIF2S1 | Eukaryotic translation initiation factor 2 subunit 1 | | -9,4 | -8,9 |
| ERN1 | Serine/threonine-protein kinase/endoribonuclease IRE1 | | -1,7 | -0,9 |
| ERO1L | ERO1-like protein alpha | | 5,9 | 2,6 |
| EXTL3 | Exostosin-like 3 | | -3,1 | -3,4 |
| FAM129A | Protein Niban | | 2,1 | 3,1 |
| FKBP14 | Peptidyl-prolyl cis-trans isomerase FKBP14 | | -1,4 | -1,3 |
| GFPT1 | Glucosamine--fructose-6-phosphate aminotransferase [isomerizing] 1 | | -1,1 | -1,0 |
| GOSR2 | Golgi SNAP receptor complex member 2 | | -3,9 | -5,3 |
| GSK3A | Glycogen synthase kinase-3 alpha | | -5,4 | -4,7 |
| GSK3B | Glycogen synthase kinase-3 beta | | -1,7 | -1,8 |
| HDGF | Hepatoma-derived growth factor | | -3,0 | -4,1 |
| HERPUD1 | Homocysteine-responsive endoplasmic reticulum-resident ubiquitin-like domain member 1 protein | | 4,7 | 5,8 |
| HSP90B1 | Endoplasmin | | -2,1 | -3,3 |
| HSPA5 | 78 kDa glucose-regulated protein | | -4,7 | -3,4 |
| HYOU1 | Hypoxia up-regulated protein 1 | | -5,1 | -7,3 |
| IL8 | Interleukin-8 | | -4,7 | 2,9 |
| KLHDC3 | Kelch domain-containing protein 3 | | 1,3 | 2,0 |
| LMNA | Prelamin-A/C | | -9,9 | -11,1 |
| MBTPS1 | Membrane-bound transcription factor site-1 protease | | -3,1 | -1,2 |
| MBTPS2 | Membrane-bound transcription factor site-2 protease | | -0,6 | -2,4 |
| NFE2L2 | Nuclear factor erythroid 2-related factor 2 | | -4,3 | -4,2 |
| OS9 | Protein OS-9 | | -4,2 | -3,9 |
| PDIA5 | Protein disulfide-isomerase A5 | | -3,5 | -2,5 |
| PDIA6 | Protein disulfide-isomerase A6 | | -0,6 | -1,7 |
| PPP1R15A | Protein phosphatase 1 regulatory subunit 15A | | -2,5 | -1,4 |
| PPP1R15B | Protein phosphatase 1 regulatory subunit 15B | | -6,3 | -6,9 |
| PPP2R5B | Serine/threonine-protein phosphatase 2A 56 kDa regulatory subunit beta isoform | | -2,8 | -2,1 |
| PREB | Prolactin regulatory element-binding protein | | -0,5 | -2,9 |
| PTPN1 | Tyrosine-protein phosphatase non-receptor type 1 | | 2,9 | 2,8 |
| SCAMP5 | Secretory carrier-associated membrane protein 5 | | 4,0 | 7,0 |
| SEC31A | Protein transport protein Sec31A | | 2,5 | 4,5 |
| SERP1 | Stress-associated endoplasmic reticulum protein 1 | | -3,9 | -1,1 |
| SHC1 | SHC-transforming protein 1 | | -3,3 | -4,7 |
| SRPR | Signal recognition particle receptor subunit alpha | | -2,8 | -2,4 |
| SRPRB | Signal recognition particle receptor subunit beta | | -6,3 | -7,2 |
| SSR1 | Translocon-associated protein subunit alpha | | -2,7 | -1,4 |
| SULT1A4 | Sulfotransferase 1A3/1A4 | | 2,1 | 0,9 |
| SYVN1 | E3 ubiquitin-protein ligase synoviolin | | -3,6 | -2,4 |
| TATDN2 | Putative deoxyribonuclease TATDN2 | | -3,7 | -4,5 |
| TLN1 | Talin-1 | | -1,3 | 1,5 |
| TP53 | Cellular tumor antigen p53 | | -4,4 | -2,2 |
| TPP1 | Tripeptidyl-peptidase 1 | | 4,9 | 6,2 |
| TSPYL2 | Testis-specific Y-encoded-like protein 2 | | -3,2 | -1,6 |
| UBE4B | Ubiquitin conjugation factor E4 B | | 2,7 | 4,2 |
| USP19 | Ubiquitin carboxyl-terminal hydrolase 19 | | 0,9 | 2,7 |
| VAPB | Vesicle-associated membrane protein-associated protein B/C | | 1,6 | 4,5 |
| VCP | Transitional endoplasmic reticulum ATPase | | 8,4 | 5,8 |
| WFS1 | Wolframin | | 9,9 | 9,1 |
| WIPI1 | WD repeat domain phosphoinositide-interacting protein 1 | | 10,7 | 11,0 |
| XBP1 | X-box-binding protein 1 | | -0,7 | 1,0 |
| YIF1A | Protein YIF1A | | -6,2 | -5,2 |
| YOD1 | Ubiquitin thioesterase OTU1 | | -1,2 | -1,7 |
| ZBTB17 | Zinc finger and BTB domain-containing protein 17 | | -5,5 | -4,0 |

Supplementary Table 1
